# Evidence-based medicine and clinical fluorodeoxyglucose PET/MRI in oncology

**DOI:** 10.1186/s40644-015-0053-1

**Published:** 2015-11-17

**Authors:** Kenneth Miles, Liam McQueen, Stanley Ngai, Phillip Law

**Affiliations:** Department of Diagnostic Imaging, Princess Alexandra Hospital, Woolloongabba, Brisbane, Australia; Institute of Nuclear Medicine, University College London, London, UK; Department of Health, Health Technology Assessment & Evaluation, Queensland Government, Herston, Brisbane, Australia

## Abstract

Positron Emission Tomography/Magnetic Resonance Imaging (PET/MRI) is a hybrid of two technologies each with its own evidence for clinical effectiveness. This article amalgamates evidence for clinical effectiveness of fluorodeoxyglucose (FDG) PET/CT and MRI as separate modalities with current evidence for hybrid PET/MRI and considers whether such an approach might provide a stronger case for the clinical use of PET/MRI at an earlier stage.

Because links between diagnostic accuracy and health outcomes have already been established for FDG-PET/CT in the investigation of suspected residual or recurrent malignancies, evidence showing improved diagnostic performance and therapeutic impact from the use of PET/MRI as an alternative would imply clinical effectiveness of this modality for this application. A meta-analysis of studies comparing FDG-PET/CT to MRI in patients with suspected residual disease or recurrence of tumours indicates complementary roles for these modalities. PET demonstrates greater sensitivity for recurrence within lymph nodes whereas MRI is more effective that PET/CT in the detection of skeletal and hepatic recurrence. A review of studies assessing therapeutic impact of PET/MRI suggests a greater likelihood for change in clinical management when PET/MRI is used for assessment of suspected residual or recurrent disease rather than tumour staging.

Supplementing the evidence-base for FDG-PET/MRI with studies that compare the components of this hybrid technology deployed separately indicates that FDG-PET/MRI is likely to be clinical effective for the investigation of patients with a range of suspected residual or recurrent cancers. This indication should therefore be prioritised for further health technology assessment.

## Background

William Osler’s description of medicine as “a science of uncertainty and an art of probability” is as pertinent now as it was in his time [1]. A frequent area of uncertainty in medicine today relates to the introduction of emerging health technologies, where early adoption is often associated with lack of clarity regarding the clinical applications that add value for patients and society. This situation reflects the current status of Positron Emission Tomography/Magnetic Resonance Imaging (PET/MRI) in oncology. The ability to simultaneously acquire PET images of tissue function and MRI images of soft tissue morphology represents a technological advance on PET/Computed Tomography (CT) which is currently used widely for staging and assessment of suspected residual or recurrent disease in patients with a range of tumour types.

Whilst recognising the potential for PET/MRI to impact on priority health areas, recent technology briefs in Australia and the UK have both highlighted the need for better evidence of clinical effectiveness for this technology [2, 3]. Emerging technologies typically have a limited evidence-base from which to identify clinical applications and accumulation of sufficient evidence to justify clinical use may take a significant amount of time. However, hybrid devices such as PET/MRI do not represent a completely new technology, but integrate two pre-existing technologies each with its own evidence for clinical effectiveness. A different approach to evidence synthesis that assimilates data for PET/CT and MRI as separate modalities could therefore potentially supplement the currently limited evidence derived using hybrid PET/MRI systems. This article considers whether such an approach might provide a stronger case for the clinical use of PET/MRI at an earlier stage.

### Technical advantages of PET/MRI

An important technical advantage for PET/MRI is a reduction radiation exposure for patients. With PET/CT, the CT component is used for attenuation correction, and for the localisation and characterisation of lesions identified by PET. Although attenuation correction is achieved using a low-dose protocol, additional diagnostic quality CT acquisitions may be required for accurate lesion localisation and characterisation. If these CT acquisitions are replaced by MRI, it can be estimated that dose reductions of 1.5 to 19.4 mSv per examination could be achieved [4]. There are also opportunities to reduce the amount of radiotracer administered.

Secondly, the high tissue contrast afforded by MRI offers the potential to compensate for some diagnostic limitations of PET/CT, such as the constraints created by background physiological tracer uptake in certain organs. For the most commonly used clinical PET tracer, Fluorodeoxyglucose (FDG), the reduced sensitivity of PET for cerebral metastases due to high physiological radiotracer uptake is sufficiently well recognised for brain MRI to be frequently included in current care pathways. However, there is also significant physiological FDG uptake in liver and bone marrow and addition of appropriate MRI sequences to FDG-PET could similarly improve detection of metastatic disease in these organs. Appropriate whole-body MRI acquisitions for detection of skeletal metastases can be acquired concurrently with the whole-body PET images. For maximal detection of liver lesions, an additional dedicated series of acquisitions in a single bed-position can be readily appended to the whole-body acquisitions, including images with liver-specific contrast material [5]. The multiple MR sequences acquired in this way can also potentially improve tissue characterisation in comparison to CT, for example aiding the distinction between malignant and inflammatory causes of FDG uptake.

### Advantages of PET/MRI over separately acquired PET/CT and MRI

An important consideration for clinical PET/MR is the extent to which simultaneous PET/MR benefits over sequential PET/CT and MRI acquisitions with either side-by-side interpretation or software fusion. Acquiring PET and MRI data sets on a single device avoids duplication of booking procedures, saves time and number of departmental visits for the patient, and avoids the radiation dose associated with the low-dose CT component of PET-CT. Simultaneous acquisition of PET and MR images results in highly accurate anatomical registration that is more readily integration into clinical workflow than software co-registration of separately acquired PET/MRI data sets. Furthermore, software fusion approaches cannot reliably compensate for the differences in respiration, peristalsis, and filling of bowel and bladder that frequently occur between images of thorax, abdomen and pelvis acquired at different times [6]. Image co-registration afforded by PET/MRI is also superior to that achievable with PET/CT devices which acquire the image datasets sequentially, albeit in close temporal proximity on the same imaging table, resulting in greater confidence in assignment of areas of radiotracer uptake to anatomical findings [7]. Studies comparing side-by-side interpretation with integrated image acquisition for other hybrid imaging modalities such as SPECT/CT and PET/CT have confirmed that more accurate co-registration of functional and anatomical datasets is associated with greater diagnostic specificity and fewer indeterminate reports and such benefits can be also be anticipated for PET/MRI [8, 9]. There are also opportunities for PET/MR to improve quantification of radiotracer uptake by more accurate compensation for body composition and/or intra-lesional fat, analogous to methods proposed for brain PET/MRI [10].

### Patient groups who may benefit from PET/MRI

Reductions in radiation exposure due diagnostic imaging would be a particular advantage for paediatric patients. However, as the incidence of cancer in this population is relatively low, the burden of disease is unlikely to justify installation of PET/MR outside highly specialised centres. A second group of patients for whom radiation exposure from diagnostic tests is emerging is a significant issue comprises cancer survivors. A recent study has estimated the risk of second cancer induction by the use of CT in this group of patients to be between 0.1 and 10 % [11]. With the growing success of first-line cancer therapy leading to an increasing population of cancer survivors, survivorship is emerging as a significant challenge for health care. For example, it has been estimated that 13.7 million cancer survivors were living in the US as of January 2012 as compared to 1.6 million new cancer cases diagnosed in the US that year, a ratio greater than 8:1 [12]. Diagnostic imaging, including PET/CT, is frequently used in cancer survivors when residual or recurrent disease is suspected on the basis of symptoms or rising tumour markers. If tumour is excluded, patients can continue routine surveillance. If residual or recurrent tumour is confirmed, patients with localised disease may benefit from surgery or other local therapy such as stereotactic ablative radiotherapy, whereas second-line chemotherapy or best-supportive care would be appropriate for patients with extensive disease. Alternatively, imaging instigated for suspected recurrence may also reveal a second malignancy. In this clinical context, residual disease needs to be distinguished from post-treatment inflammatory change whilst the brain, liver and bone marrow are common sites for disease recurrence. Thus, this group of patients could also benefit from the potential for PET/MRI to overcome current limitations of PET/CT in this clinical context.

### Evaluating evidence for clinical effectiveness of hybrid imaging technologies

Clinical effectiveness of a diagnostic test is defined by the extent to which incorporating the test into clinical practice improves health outcomes [13]. Direct evidence of the impact of a diagnostic test on health outcomes is rarely available due to a range of methodological difficulties. Clinical effectiveness can therefore also be demonstrated by using evidence that links test accuracy with evidence that the test result changes treatment practice, and with evidence that the alternative treatments have different effectiveness and safety profiles [13]. Links between diagnostic accuracy and health outcomes have already been established for a range of clinical applications for PET and MRI as separate modalities. For investigation of suspected residual or recurrent malignancies, strong links have been established between FDG-PET and clinical outcome for patients with lymphoma, sarcoma, malignant melanoma and cancers of the colon or rectum, ovary, uterine cervix, and head & neck. The key issues for the clinical effectiveness of hybrid PET/MRI devices in the assessment of suspected residual or recurrent malignancy are therefore a) the extent to which combining these modalities can improve diagnostic performance compared to either modality alone, and b) whether any improvements diagnostic performance lead to changes in treatment practice.

### Comparative studies of diagnostic accuracy of FDG-PET and MRI for residual/recurrent malignancy

The Cochrane Collaboration has stipulated standards for the analysis of comparative studies of diagnostic accuracy [14]. To minimise bias, such studies employ a direct comparison of the tests in question by either applying both tests to each individual, or randomising each individual to receive one of the tests. A common reference standard should be consistently applied to both tests. Nine studies that meet these standards whilst reporting the accuracy of MRI and FDG-PET in patients with suspected residual or recurrent disease for the tumour types listed above are available [15–23].

#### Detection of tumour recurrence within lymph nodes, bone and liver

Seven publications have reported the diagnostic performances of PET/CT and MRI in the detection of nodal, skeletal or hepatic metastases in patients clinically suspected to have recurrent tumours [15–21]. Three of these reports considered patients with recurrent melanoma (total number of patients = 136), one study comprised patients with recurrent colorectal (24 patients), one study considered patients with head and cancer (179 patients) and 2 studies included patients with various non-central nervous system tumours (total number of patients = 72). For each study the reference standard consisted of histology or clinical follow-up for at least 6 months.

**Fig. 1:**
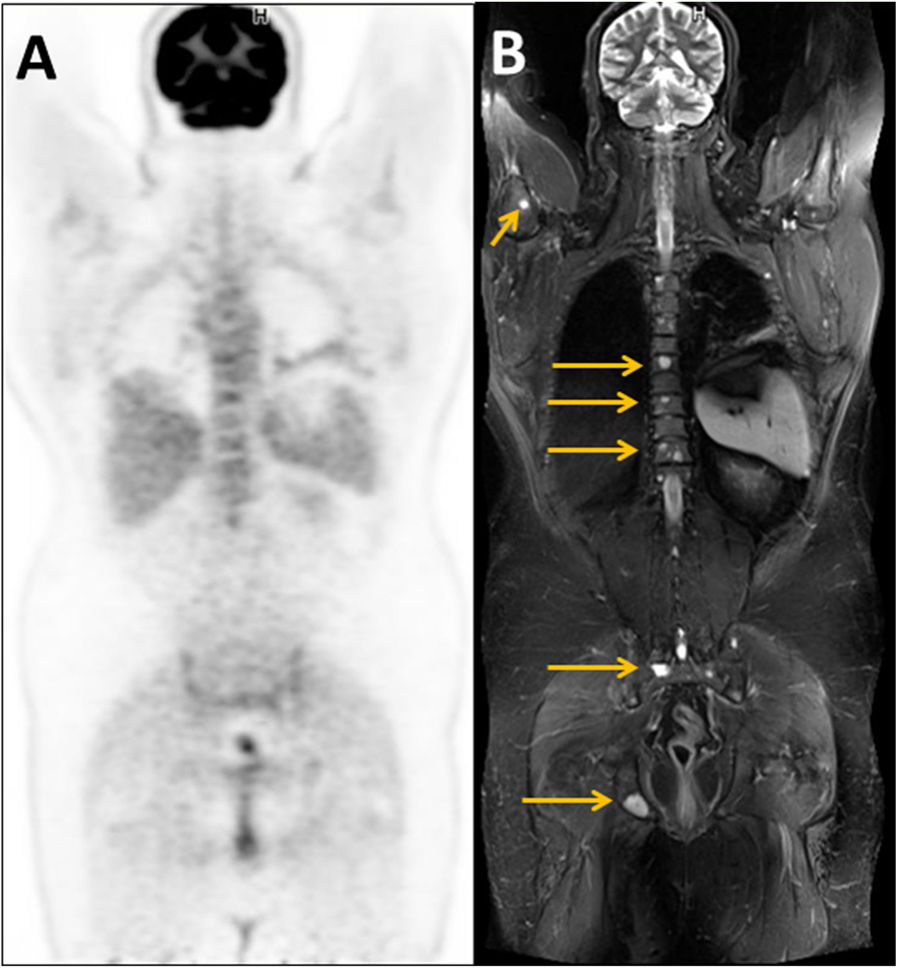
PET/MRI study comprising whole-body FDG-PET (**a**) and Short Tau Inversion Recovery (STIR) MRI (**b**) from a patient with recurrent myxoid liposarcoma. The skeletal metastases are more readily appreciated on the MRI than on FDG-PET

These studies indicate complementary roles for PET and MRI in identification of tumour recurrence. FDG-PET/CT demonstrates superior diagnostic performance for recurrence in lymph nodes whilst recurrences in the skeleton and liver are more reliably depicted by MRI (Fig. 1). The weighted averages from the 5 studies reporting diagnostic performance for detection of nodal recurrence (Table 1) show superior sensitivity for PET/CT with no significant change in positive predictive value (PPV) whereas the weighted averages of the 5 studies reporting diagnostic performance for detection of skeletal recurrence (Table 2) and the 5 studies comparing PET/CT and MRI in the detection of tumour recurrence within the liver (Table 3) both confirm superior sensitivity for MRI with no significant difference in PPV.

**Table 1:** Summary of the results of test accuracy in studies comparing whole-body MRI with FDG-PET/CT in the detection of sites of tumour recurrence within lymph nodes

Study	Population & setting	Reference standard	Outcome [95 % CI]			
			PET-CT		WB-MRI	
			Sensitivity	PPV	Sensitivity	PPV
Pfannenberg et al. (2007) [15]	Recurrent melanoma	Histology or 8 months follow-up	85 [77–91] %	94 % [87–97] %	66 [56–74] %	84 % [74–90] %
	Self-controlled study (*n* = 64)					
Schmidt et al. (2009) [16]	Recurrent Colorectal Cancer	Follow-up (mean 11 months)	93 [78–98] %	100 [86–100] %	62 [44–77] %	82 [61–93] %
	Self-controlled study (*n* = 24)					
Ng et al. (2010) [18]	Recurrent Head & Neck Cancer	Histology or 12 months follow-up	80 [61–91] %	77 [58–89] %	88 [70–96] %	81 [63–92] %
	Self-controlled study (*n* = 179)					
Laurent et al. (2010) [19]	Recurrent melanoma	Histology or 6 months follow-up	83 [65–92] %	100 [86–100] %	90 [74–96] %	96 [82–99] %
	Self-controlled study (*n* = 35)					
Jouvet et al. (2014) [20]	Recurrent melanoma	Histology or ≥6 months follow-up	96 [79–99] %	96 [79–99] %	87 [68–95] %	100 [84–100] %
	Self-controlled study (*n* = 37)					
*Weighted averages*	*(n = 339)*		*87 [81–91] %*	*93 [89–96] %*	*74 [67–79] %*	*87 [81–91] %*

**Table 2:** Summary of the results of test accuracy in studies comparing whole-body MRI with FDG-PET/CT in the detection of sites of skeletal metastases

Study	Population & setting	Reference standard	Outcome [95 % CI]			
			PET-CT		WB-MRI	
			Sensitivity	PPV	Sensitivity	PPV
Pfannenberg et al. (2007) [15]	Recurrent melanoma	Histology or 8 months follow-up	91 [78–97] %	91 % [78–97] %	100 [90–100] %	90 % [76–96] %
	Self-controlled study (*n* = 64)					
Schmidt et al. (2007) [16]	Non-CNS tumours and suspicion of bone metastases	≥6 months imaging follow-up	77 [68–84] %	94 [67–97] %	94 [88–97] %	94 [88–97] %
	Self-controlled study (*n* = 35)					
Schmidt et al. (2009) [17]	Recurrent Colorectal Cancer	Follow-up (mean 11 months)	50 [15–85] %	100 [51–100] %	100 [51–100] %	100 [51–100] %
	Self-controlled study (*n* = 24)					
Laurent et al. (2010) [19]	Recurrent melanoma	Histology or 6 months follow-up	71 [45–88] %	100 [72–100] %	93 [69–99] %	100 [77–100] %
	Self-controlled study (*n* = 35)					
Jouvet et al. (2014) [20]	Recurrent melanoma	Histology or ≥6 months follow-up	88 [64–97] %	93 [70–99] %	100 [81–100] %	76 [55–89] %
	Self-controlled study (*n* = 37)					
*Weighted averages*	*(n = 195)*		*80 [74–85] %*	*94 [89–97] %*	*96 [92–98] %*	*92 [87–95] %*

**Table 3:** Summary of the results of test accuracy in studies comparing whole-body MRI with FDG-PET/CT in the detection of sites of tumour recurrence within the liver

Study	Population & setting	Reference standard	Outcome [95 % CI]			
			PET-CT		WB-MRI	
			Sensitivity	PPV	Sensitivity	PPV
Donati et al. (2010) [21]	Various (*n* = 37, Colorectal Cancer: *n* = 20)	Histology or follow-up	76 [64–86] %	93 [82–98] %	91 [80–96] %	100 [93–100] %
Schmidt et al. (2009) [16]	Recurrent Colorectal Cancer	Follow-up (mean 11 months)	86 [65–95] %	100 [82–100] %	100 [85–100] %	100 [85–100] %
	Self-controlled study (*n* = 24)					
Pfannenberg et al. (2007) [15]	Recurrent melanoma	Histology or 8 months follow-up	94 [81–98] %	100 [90–100] %	100 [90–100] %	100 [90–100] %
	Self-controlled study (*n* = 64)					
Laurent et al. (2010) [19]	Recurrent melanoma	Histology or 6 months follow-up	50 [15–85] %	100 [51–100] %	100 [51–100] %	100 [51–100] %
	Self-controlled study (*n* = 35)					
Jouvet et al. (2014) [20]	Recurrent melanoma	Histology or ≥6 months follow-up	100 [76–100] %	100 k[76–100] %	100 [76–100] %	92 [67–99] %
	Self-controlled study (*n* = 37)					
*Weighted averages*	*(n = 90)*		*84 [77–90] %*	*97 [92–99] %*	*96 [91–98] %*	*99 [96–100] %*

#### Identification of patients with tumour recurrence

Three studies compared the performance of FDG-PET/CT to combined reading of PET/CT and MRI in patients with suspected tumour recurrence (Table 4). The first comprised a meta-analysis which identified 4 studies that comparing the diagnostic performance on a per-patient basis of PET/CT alone to PET combined with WB-MRI in the detection of residual or recurrent tumour of the head and neck [22]. Combined reading improved the sensitivity for the identification of patients with residual or recurrent disease from 82 % (95 % CI: 69–90 %) to 89 % (95 % CI: 86–96 %) with no loss of specificity (PET-CT: 97 [94–98]%; combined reading: 98 [97–99]%). Additional to the above meta-analysis, in a study of 67 patients undergoing both dedicated PET/MRI and PET/CT, Biederwellen et al. [23] reported a trend for improved sensitivity when using PET/MRI (100 %, [95 % CI: 72–100 %] versus 90 % [68–98 %] for PET/CT) with no change in specificity (each 100 %; [94–100]%). On the other hand, a study of 37 patients by Donati et al. found no significance difference in the ability of retrospectively fused PET and MRI data sets compared to PET-CT to identify patients with liver metastases [21].

**Table 4:** Summary of the results of test accuracy in studies comparing combined MRI and FDG-PET/CT to PET/CT alone for the classification of patients with or without tumour recurrence

Study	Population & setting	Reference standard and comparator	Outcome [95 % CI]			
			PET/CT		PET/MR	
			Sensitivity	Specificity	Sensitivity	Specificity
Xu et al. (2013) [22]	Meta-analysis of 4 studies comparing PET/CT and WB-MRI in the detection metastatic head & neck cancer (*n* = 511)	Reference standard variable.	82 [69–90] %	97 [94–98] %	89 [86–96] %	98 [97–99] %
		Combined reading of PET and WB-MRI				
Donati et al. (2010) [21]	Hepatic metastases (*n* = 37, CRC: *n* = 20)	Histology or follow-up Fused PET/MR	100 [77–100] %	92 [67–99] %	100 [77–100] %	100 [77–100] %
Beiderwellen et al. (2014) [23]	Skeletal metastases (*n* = 67)	Histology or follow-up	90 [68–98] %	100 [72–100] %	100 [94–100] %	100 [94–100] %
		Dedicated PET/MR				

### Changes in treatment practice from FDG-PET/MRI for patients suspected residual or recurrent malignancy

Three studies have reported therapeutic impact from the use of PET/MRI in place of PET/CT [7, 24, 25] (Table 5). Two of these studies [24, 25] included patients with suspected residual disease or recurrence whereas the study by Al-Nabhani et al. [7] comprised only patients for staging. The likelihood of PET/MRI detecting additional findings not identified on PET/CT with impact on clinical management is much greater in the studies including patients with suspected recurrence (Odds ratio 14.4 or infinity versus 2.1). The largest of these studies (Catalano et al.) also reported the incidence of change in management for different categories of additional finding. Change in clinical management was significantly more likely when additional findings related to recurrent or residual disease (Odds ratio calculated from reported data = 10.4, 95 % CI 2.9 – 37, *p* <0.0001).

**Table 5:** Summary table for studies reporting therapeutic impact

Study	Population	Outcome	New Technology n with event/N (%)	Comparator n with event/N (%)	Effect size (95 % CI/*p* value)
Catalano et al. (2013) [24]	Cohort observational study. Staging or follow-up of non-CNS tumours (*n* = 134)	Patients additional findings not identified on alternate modality	6/134 (4.5 %)	55/134 (41 %)	OR: 14.9 (6.1–36.1 *p* <0.0001)
		Patients with additional findings not identified on alternate modality affecting clinical management	24/134 (17.9 %)	2/134 (1.5 %)	OR: 14.4 (3.3–62.3 *p* <0.0001)
Reiner et al. (2014) [25]	Cohort observational study. Hepatic metastases (*n* = 55, CRC: *n* = 41)	Patients additional findings not identified on alternate modality	8/55 (9.1 %)	0/55 (0 %)	OR: Infinity OR: Infinity
		Patients with additional findings not identified on alternate modality affecting clinical management	5/55 (9.1 %)	0/55 (0 %)	
Al-Nabhani et al. (2014) [7]	Cohort observational study. Staging of non-CNS tumours (*n* = 50)	Patients with additional findings not identified on alternate modality affecting clinical management	4/50 (8 %)	1/50 (2 %)	OR: 2.1 (0.36–11.9 NS)

### The need for further clinical evaluation of PET/MRI in residual/recurrent malignancy

There are areas in which the evidence-based for PET/MRI in the assessment of suspected residual or recurrent malignancy is deficient. Current management of residual/recurrent tumour requires stratification of patients beyond the presence or absence of tumour. Local treatments such as surgery or radiotherapy may be appropriate for patients with localised recurrence whereas systemic treatment such as chemotherapy or palliative treatment would be most appropriate for disseminated disease. Using the available literature, it has not been possible to assess the potential impact of PET/MRI over PET/CT in stratifying patients as described above and we highlight the need for such studies in future. Furthermore, even after the evidence-synthesis described in our study, current literature is not sufficiently mature to enable an assessment of whether the implied effectiveness of PET/MRI in recurrent/residual malignancy is likely to be cost-effective. The capital cost of PET/MRI devices are comparable to PET/CT and MRI systems purchased separately and therefore, to ensure cost-effectiveness, PET/MRI workflows need to minimise the amount of time when either component is idle. Where PET/MRI replaces PET/CT and MRI performed separately, streamlining of clerical, radiographer and nursing work related to imaging investigation of patients with residual or recurrent cancer into a single imaging episode may defray some of these costs. On the other hand, potential improvements in cost-effectiveness can be anticipated. The therapeutic impact study of Catalano et al. found the commonest change in management when PET/MRI was used for patient with residual or recurrent disease was avoidance of biopsy [24]. Further management changes of potential health economic importance include avoiding the cost and morbidity of futile local treatments that would have been inappropriately selected due to under-estimation of disease extent by current technology, earlier identification of limited or disseminated disease allowing timely instigation of local therapy or salvage therapy respectively, and avoidance of futile chemotherapy in the presence of advanced disease of an extent under-estimated by current technology. Future studies are also needed to address these aspects of PET/MRI deployment.

Other potential benefits of PET/MRI are less tangible than those that can be inferred from improved diagnostic performance and therapeutic impact over PET/CT. Combining into a single procedure, examinations that in some instances may have been performed on separate devices and/or separate occasions using current technology, will result in increased convenience for patients such as reduced travel costs and fewer attendance days, with more rapid availability of the results of imaging assessment allowing earlier clinical decision making. Deployment of PET/MRI could also usefully increase capacity for MRI at a time when utilisation of MRI for oncological applications is increasing [26]. The impact of the reduced radiation exposure for patients from the use of MRI in place of CT can also be anticipated to improve health outcomes by reducing the risks of second cancer induction which have been shown to be significant for cancer survivors [11]. However, such benefits are harder to quantify in health economic terms and are not typically included in economic analyses of diagnostic imaging technologies. The impact of reduced risk of adverse reaction to contrast material for those situations where PET/MRI has replaced PET/CT with contrast enhancement is similarly hard to quantify.

The approach to evidence-based synthesis outlined above for FDG-PET is currently not applicable for other PET tracers with emerging roles in oncology such as ^68^Ga-dotatate and ^68^Ga-Prostate Specific Membrane Antigen because the evidence-based for these tracers is underdeveloped in comparison to FDG. In particular, there is a paucity of data linking the use of these tracers with improvements in health outcomes and few studies comparing their use with PET/CT against whole-body MRI. Therefore, the clinical effectiveness of these emerging tracers will need to be evaluated using conventional approaches to health technology assessment approaches.

### Considerations for PET/MRI service delivery

There are a range of further issues that would need to be addressed before implementing clinical PET/MRI for evaluation of suspected residual or recurrent disease. Firstly, there would need to be sufficient patients with relevant clinical need. Secondly, PET/MRI would need to co-located with PET/CT to ensure available of PET for patients with contraindications to MRI (e.g., MR incompatible implantable medical devices, previous ocular metallic foreign body) and to meet the circumstances when PET/CT may be superior to PET/MRI, for example the visualisation of small pulmonary nodules. Overall imaging demand would therefore need to justify 2 PET systems (one PET/MRI and one PET/CT) and thus a regional oncology centre would likely be the most appropriate location for such a service. Even under these circumstances, PET/MRI research and/or additional standalone MR examinations would likely be needed to completely fill the capacity of the PET/MRI installation. Although the clinical component of such a combined program may be based on a remote supply of FDG, colocation with a cyclotron would facilitate a parallel PET/MR research program by making other PET tracers available for oncological research. Furthermore, the clinical and research components of such a programme would each require availability and co-ordination of technical and radiological expertise in both nuclear medicine and oncologic MRI. Funding of a clinical FDG-PET/MRI service may also be problematic as, depending on the relevant health-care system, if accessible at all, re-imbursement may only be available at the same level as PET/CT. Studies demonstrating the cost-effectiveness of PET/MR in comparison to PET/CT may be required before a level of reimbursement reflecting the additional cost associated with PET/MRI becomes available.

## Conclusion

This review has illustrated two issues related to evidence-based assessments of hybrid imaging technologies such as PET/MRI. Firstly, the requirement to demonstrate a link between diagnostic performance and health outcomes may have already been met by the existing evidence-base for either component of the hybrid technology deployed alone. In the case of FDG-PET/MRI for patients with suspected recurrent or residual malignancy, such links have already been demonstrated for the PET component for a range of tumour types. Secondly, rather than rely solely on technology assessments of the new device, the evidence-base for hybrid technologies such as PET/MRI can be supplemented by studies comparing the components of the hybrid technology deployed separately. In the current study, this approach to evidence-synthesis has identified additional support for the application of PET/MRI in the assessment of suspected recurrent of residual malignancy by providing supplementary evidence of improved detection of malignant lesions in bone and liver whilst preserving the effectiveness of PET for lymph node recurrence. Furthermore, a review of studies assessing therapeutic impact of FDG-PET/MRI suggests a greater likelihood for change in clinical management when FDG-PET/MRI is used for assessment of suspected residual or recurrent disease rather than tumour staging. Thus, supplementing the evidence-base for FDG-PET/MRI with studies that compare the components of this hybrid technology deployed separately suggests that FDG-PET/MRI is likely to be clinical effective for the investigation of patients with a range of suspected residual or recurrent cancers. This indication should therefore be prioritised for further health technology assessment.
